# Supplementary material for: What drives willingness to receive a new vaccine that prevents an emerging infectious disease? A discrete choice experiment among university students in Uganda
Source: PLoS One. 2022 May 19;17(5):e0268063. doi: 10.1371/journal.pone.0268063 (PMC9119467; doi:10.1371/journal.pone.0268063)
Supplement: S1 Table — (DOCX) [file pone.0268063.s001.docx]

### Supplementary materials

S1 illustrates the number and percentage of students that met the criteria for each of the sensitivity tests; the number of excluded students from each group; the risk difference in willingness to accept a vaccine between groups; and the risk difference in answering consistently answering the duplicate questions between groups. Overall, 94% of students answered the survey in more than eight minutes, 87% of students answered the two duplicate survey questions consistently, 88% of students self-reported considering at least three of the six attributes always or sometimes. We observed that the risk difference in vaccination willingness between students in health disciplines and other students was consistent across sensitivity tests and that the difference in administration routes was small and not significant.

| S1. Sensitivity test by number, proportion, and predicted probability of difference in vaccination willingness between students in health disciplines and students in other disciplines | | | | | |
| --- | --- | --- | --- | --- | --- |
| Sensitivity test* | Passed sensitivity test^τ^n(%) | Health disciplines excluded n (%) | Other disciplines excluded n % | Willingness  risk difference^ϒ^ (95% CI) | Duplicates  risk difference^ι^ (95% CI) |
| Full dataset | 1576 (100) | 0 (100) | 0 (100) | 6.72 (4.1-9.3) | 78.0 (76.8-79.2)^ω^ |
| 1.Survey time | 1,484 (94.2) | 71 (9.1) | 21 (2.7) | 6.9 (4.3-9.7) | - |
| 2. Duplicates | 1,371 (87.0) | 90 (11.5) | 115 (14.5) | 6.1 (3.4- 8.7) | - |
| 3.Attributes considered | 1,387 (88.0) | 81 (10.3) | 108 (13.6) | 6.3 (3.7- 9.0) | - |
| 4.Administration^±^ | 659 (41.8) | 775 (99.0) | 142 (17.9) | - | 0.0 (<0.01-<0.01) |
| ^*^Sensitivity tests included the following criteria: Survey time defined as survey time >8 min (yes/no); Duplicates defined as a consistent answer to the two duplicate survey questions (yes/no); Attributes considered defined as considering at least three of the six attributes in the survey sometimes or always.  ^τ^Passing a sensitivity test indicates that the students’ survey responses were within the categories listed above. Students with responses that “passed” a sensitivity test were included in the sensitivity analyses.  ^ϒ^ Dataset restricted to only individuals who “passed” their respective sensitivity test. We compared the difference in vaccination willingness between students in health disciplines and students from other disciplines (referent) using a panel mixed logit model, adjusting for covariates described in methods, and reported the risk difference.  ^ι^ We compared the consistency in responses to the duplicate questions between those who received a choice of administration compared to interviewer administered surveys. We use a panel mixed logit model with a covariate for administration route, adjusted for covariates described, and reported the risk difference between groups.  ^±^Administration route defined as either giving students a choice of self or interviewer-administered surveys or solely interviewer-administered surveys.  ^ω^This is the predicted probability of vaccination willingness in the full analytic dataset.  Supplementary Table 2 illustrates the odds ratios of the attribute specific parameters associated with willingness to receive a new vaccine for the entire analytic dataset as well as each of the four sensitivity tests: survey time >8 minutes, consistently answering the duplicate questions, varying responses across the panel of questions, and considering three or more attributes at least sometimes when completing the survey. We see that subgroup of participants included in each of these sensitivity tests had similar preferences to the participants in the full analytic dataset. | | | | | |
