# Supplementary material for: What drives willingness to receive a new vaccine that prevents an emerging infectious disease? A discrete choice experiment among university students in Uganda
Source: PLoS One. 2022 May 19;17(5):e0268063. doi: 10.1371/journal.pone.0268063 (PMC9119467; doi:10.1371/journal.pone.0268063)
Supplement: S2 Table — (DOCX) [file pone.0268063.s002.docx]

S2: The odds ratio (OR) of attribute-specific parameters associated with willingness to receive a new vaccine, subset by sensitivity tests^t^

| PARAMETERS |  | All | Survey time | Duplicates | Straightliner | Attributes considered |
| --- | --- | --- | --- | --- | --- | --- |
|  |  | OR (95% CI) | OR (95% CI) | OR (95% CI) | OR (95% CI) | OR (95% CI) |
| **Risk – Willingness to receive new vaccine given the following risk** | |  |  |  |  |  |
| (highest risk) Someone in your current household touched an infected person | | 3.0 (2.6-3.5) | 3.0 (2.6-3.5) | 3.5 (2.9-4.3) | 3.0 (2.5-3.4) | 2.9 (2.4-3.4) |
| (higher risk) 2 cases of the disease were just reported in your district | | 1.5 (1.3-1.8) | 1.6 (1.3-1.8) | 1.6 (1.3-1.9) | 1.5 (1.3-1.8) | 1.5 (1.3-1.8) |
| (low risk) 2 cases of the disease were just reported in a distant region of Uganda | | 1.1 (0.9-1.2) | 1.1 (0.9-1.3) | 1.2 (1.0-1.4) | 1.1 (0.9-1.3) | 1.0 (0.9-1.2) |
| (lowest risk) 25 cases of the disease were just reported in a neighboring country | | ref | ref | ref | ref | ref |
| **Severity** |  |  |  |  |  |  |
| (highest risk) The disease kills 50% (5 in 10) of people infected | | 5.0 (4.2-5.8) | 5.2 (4.4-6.1) | 4.7 (3.9-5.7) | 4.7 (4.0-5.5) | 5.2 (4.4-6.2) |
| (high risk) The disease kills 10% (1 in 10) of people infected | | 2.4 (2.1-2.8) | 2.4 (2.1-2.8) | 2.4 (2.0-2.8) | 2.3 (2.0-2.6) | 2.6 (2.2-3.0) |
| (low risk) The disease kills 1% (1 in 100) of people infected | | 1.4 (1.3-1.7) | 1.4 (1.2-1.7) | 1.4 (1.2-1.6) | 1.4 (1.2-1.6) | 1.5 (1.3-1.7) |
| (lowest risk) The disease kills 0.1% (1 in 1,000) of people infected | | ref | ref | ref | ref | ref |
| **Trusted Individuals** |  |  |  |  |  |  |
| A family member or friend that you trust advised you to take the vaccine | | 1.9 (1.6-2.2) | 1.9 (1.6-2.2) | 2.3 (1.9-2.7) | 1.9 (1.6-2.2) | 1.9 (1.6-2.2) |
| A religious or tribal leader that you trust advised you to take the vaccine | | 1.6 (1.3-1.8) | 1.6 (1.3-1.8) | 1.8 (1.5-2.2) | 1.6 (1.4-1.8) | 1.5 (1.3-1.8) |
| A religious or tribal leader that you trust advised you not to take the vaccine | | 1.1 (0.9-1.3) | 1.1 (0.9-1.3) | 1.2 (1.0-1.4) | 1.1 (1.0-1.3) | 1.1 (0.9-1.3) |
| A family member or friend that you trust advised you not to take the vaccine | | ref | ref | ref | ref | ref |
| **Influential Voices** |  |  |  |  |  |  |
| The Minister of Health recommended that people take the vaccine | | 1.9 (1.6-2.2) | 2.3 (2.0-2.8) | 2.5 (2.1-3.1) | 2.1 (1.8-2.5) | 2.5 (2.1-3.0) |
| Your favorite social media blogger advised people to take the vaccine | | 1.6 (1.3-1.8) | 1.2 (1.0-1.4) | 1.5 (1.3-1.8) | 1.2 (1.0-1.4) | 1.2 (1.1-1.5) |
| Your favorite social media blogger advised people not to take the vaccine | | 1.1 (0.9-1.3) | 0.9 (0.8-1.0) | 1.0 (0.8-1.1) | 0.9 (0.8-1.1) | 0.9 (0.8-1.1) |
| An opposition politician warned people not to take the vaccine | | ref | ref | ref | ref | ref |
| **Vaccine Protection** |  |  |  |  |  |  |
| By getting vaccinated, you protect yourself and others | | 1.6 (1.4-1.8) | 1.7 (1.5-1.9) | 1.8 (1.6-2.1) | 1.6 (1.4-1.8) | 1.7 (1.5-1.9) |
| By getting vaccinated you protect only yourself, but not others | | ref | ref | ref | ref | ref |
| **Side Effects** |  |  |  |  |  |  |
| The vaccine gives 20% of people a skin rash somewhere on their body for 3 days | | 0.6 (0.5-0.7) | 0.5 (0.5-0.6) | 0.4 (0.3-0.5) | 0.6 (0.5-0.7) | 0.5 (0.5-0.6) |
| The vaccine gives 20% of people a high fever for 1 day | | 0.7 (0.6-0.9) | 0.7 (0.6-0.8) | 0.5 (0.4-0.6) | 0.7 (0.6-0.9) | 0.7 (0.6-0.8) |
| You’ve heard rumors about harmful side effects, but none have been confirmed | | 0.6 (0.5-0.7) | 0.6 (0.5-0.7) | 0.4 (0.4-0.5) | 0.7 (0.6-0.8) | 0.6 (0.5-0.7) |
| The vaccine injection is painful for 30 minutes | | ref | ref | ref | ref | ref |
| Log of the variance* |  | 1.1 (1.0-1.2) | 1.1 (1.0-1.2) | 1.2 (1.1-1.4) | -0.9 (-1.3-0.7) | 1.0 (0.8-1.2) |
| Sigma u |  | 1.7 (1.6-1.9) | 1.7 (1.6-1.9) | 1.9 (1.7-2.0) | 0.6 (0.5-0.7) | 1.7 (1.6-1.8) |
| rho |  | 0.5 (0.4-0.5) | 0.5 (0.4-0.5) | 0.5 (0.5-0.6) | 0.1 (0.1-0.1) | 0.5 (0.4-0.5) |
| Sex | Female | 0.8 (0.6-1.0) | 0.8 (0.6-0.9) | 0.7 (0.6-1.0) | 0.9 (0.8-1.0) | 0.7 (0.6-0.9) |
|  | Male | ref |  |  |  |  |
| Age |  | 1.0 (1.0-1.1) |  |  |  |  |
| Religion | Muslim | 0.9 (0.6-1.4) | 1.0 (0.6-1.4) | 1.0 (0.6-1.6) | 0.9 (0.6-1.1) | 1.0 (0.7-1.5) |
|  | Pentecostal | 1.4 (1.0-2.0) | 1.4 (1.0-2.0) | 1.4 (0.9-2.1) | 1.1 (0.9-1.4) | 1.5 (1.0-2.2) |
|  | Protestant | 0.9 (0.7-1.2) | 1.0 (0.7-1.2) | 0.9 (0.7-1.2) | 0.9 (0.8-1.1) | 0.9 (0.7-1.2) |
|  | Other | 0.8 (0.5-1.2) | 0.7 (0.5-1.1) | 0.8 (0.5-1.2) | 0.9 (0.7-1.2) | 0.8 (0.5-1.3) |
|  | Catholic | ref |  |  |  |  |
| Region of birth | Western | 1.2 (1.0-1.6) | 1.2 (0.9-1.6) | 1.2 (0.9-1.7) | 1.1 (0.9-1.3) | 1.3 (1.0-1.7) |
|  | Northern | 1.1 (0.7-1.7) | 1.2 (0.7-1.9) | 1.1 (0.6-1.8) | 1.1 (0.8-1.4) | 1.2 (0.7-1.9) |
|  | Eastern | 1.6 (1.2-2.1) | 1.5 (1.1-2.1) | 1.5 (1.1-2.2) | 1.2 (1.0-1.5) | 1.6 (1.1-2.1) |
|  | Outside Uganda | 0.6 (0.3-1.2) | 0.6 (0.3-1.1) | 0.5 (0.3-1.1) | 0.8 (0.5-1.1) | 0.6 (0.3-1.1) |
|  | Central | ref |  |  |  |  |
| Hepatitis B vaccine | Yes | 2.0 (1.6-2.5) | 2.0 (1.6-2.4) | 2.3 (1.8-3.0) | 1.2 (1.1-1.4) | 1.8 (1.5-2.3) |
|  | No | ref |  |  |  |  |
| Number of participants | 1,574 | 1,576 | 1,142 | 1,369 | 948 | 1,385 |

Sensitivity tests included the following criteria: Survey time defined as survey time >8 min (yes/no); Duplicates defined as a consistent answer to the two duplicate survey questions (yes/no); Straightliner defined as individuals with uniform responses (always *yes* or always *no*) across the nine choice tasks; Attributes considered defined as considering at least three of the six attributes in the survey sometimes or always.

^t^This model used panel mixed logistic regression and include covariates for sex, age, region of birth, religion, and Hepatitis B vaccination status, stratified by students in health disciplines and students from other disciplines

^*^Indicates the extent of individual-level variability, with a higher value indicating greater variability within individuals
