# Supplementary material for: What drives willingness to receive a new vaccine that prevents an emerging infectious disease? A discrete choice experiment among university students in Uganda
Source: PLoS One. 2022 May 19;17(5):e0268063. doi: 10.1371/journal.pone.0268063 (PMC9119467; doi:10.1371/journal.pone.0268063)
Supplement: Attachment — Submitted filename: PLOS_UgandaReview.docx [file pone.0268063.s003.docx]

Authors,

Thank you for the opportunity to review your submission of “ What drives willingness to receive a new vaccine that prevents an emerging infectious disease? A discrete choice experiment among university students in Uganda,” submitted as an original experiment in PLOS One. The authors have a nice rationale for this extension to the literature as well as a well-established conceptualization as to where and how this paper fits into the literature. Care was obviously taken to explain the need for a more thorough understanding of vaccine willingness, specifically in the current pandemic environment. It is also apparent that the authors took care in writing this manuscript as it reads well with almost no noticeable errors in spelling and grammar. Additionally, the analysis was quite well performed and explained in the paper. There are, however, components that could be addressed to make the paper more approachable to the reader and reduce some overall confusion on the background information of the paper itself. Thank you for the opportunity to review this manuscript and I commend the authors on the vision to push forward the newest abilities of behavioral economic demand. Of note, I am not an expert in vaccination development and willingness literature but do have a substantial background and training in the field of medication willingness and adherence. Attached are a few points of feedback to consider:

**Major edits:**

Some clarity between vaccination willingness and motivating parameters would be of benefit to understand the difference of and need for Aims 2 and 3. In the paper, the authors note that the WHO incorporates vaccination willingness into vaccination motivation, however, the paper has them separated out into two separate aims. Are these two separate components that need to be investigated separately, or like the WHO notes, are these one in the same? If they are one in the same, please give some rationale as to why they need to be explored as separate processes. Some clarity as to this decision would help clear up some confusion to the reader.

Aim 1 is stated as comparing the overall differences in health professionals and other university students in Uganda. In the study design, the authors note that participants were recruited from the 6 largest colleges at Makerere University, yet the way the schools are listed it is difficult to determine if there are 6 or 8 listed. Using a numbered list might help to separate the schools so it is more apparent due to the multiple commas and “and’s” listed.

- Relatedly, there were six schools but only five collection sites. Were all school located equal distant from these collection sites? A single sentence added to address this question would be of benefit to the methods section.

It would be of use to explain which are considered health professionals within the paper. Is health science the only school considered health professionals or are veterinary medicine students lumped into this? My understanding as a reader is that the authors are evaluating those students with some background training in medicine and the body which would seem to include veterinary medicine, but this is only my assumption. Some clarity would be beneficial to understanding what metrics were used to determine health professionals. If veterinary medicine is not included in this, it would be helpful to understand how this might impact the results for the non-health professional groups.

- Additionally, overall clarity as to “health professionals,” within the introduction should be addressed. Information about and citations for “health professionals” are used throughout the introduction, yet the study was conducted on students. The authors state that “health professionals play a critical role in promoting vaccination. Therefore, there is a need to understand what motivates health professional’s willingness as their personal…” Are the students considered health professionals? As a reader, I am confused as to the classification of the students as students or professionals and if this is the same for the citations used to reference health professionals.

Throughout the introduction, vaccination willingness is mentioned but never defined or explained for the purpose of this study. Even as the authors’ note that there is a growing body of research on vaccination willingness, this topic is not further elucidated for the reader until the end of the introduction. A brief introduction into the components of vaccination willingness earlier in the introduction would do well to help the reader understand the subtleties of this term. This is clarified with the paragraph on key attributes for the study, but it comes after all the information pertinent to this topic. Even a short list of these earlier in the paper could help add some clarity.

**Minor edits:**

The paper would benefit from some clarification as to why/how the citations for studies from different countries contribute to this specific study. Specifically, the authors note that for the Ebola vaccine acceptability was high in Nigeria and Sierra Leone, but very low in the US. Does this reflect a lower willingness in more developed countries? If so, why would this study focus on Uganda? Additionally, the authors note that Liberia had a low acceptance rate of the Ebola vaccine by healthcare works. Yet, they note that the highest vaccine hesitancy took place in Uganda when immunization managers were surveyed. Further, they cite results from a study done in France that identifies key drivers of vaccination willingness. Some clarification of these competing results would make this paper much more impactful, specifically, addressing the difference in study results for developed versus developing countries.

- Within this same framework, it would be useful for the authors to address the generalizability of these results to the larger population of Uganda, or even more widely, to the world. Around 75% of Uganda residents live on less than $2 a day. Is the sample population relevant to the larger population? Does surveying those at university inform vaccination willingness for the broader scope of residents of the country? Either way this topic would be important to note for readers and researchers. It seems like comparing the college population to the non-college population might have differences that are reflective of evaluating health professionals versus non-health professionals
- The citations used for other country vaccination willingness was mostly looking at the general population, however, this study exclusively evaluated educated and therefore mostly likely higher SES samples. Does this influence the results or how it may generalize to the overall population in Uganda or other countries?

In the results, there are certain parameters, such as Influential voices, that increase willingness for non-health professionals more so than the health professionals. The overall result was reported, but information as to why this was seen could be of interest to researchers. Does background/education in health sciences decrease the importance of influential voices on the willingness to take a vaccine? Though it is at odds with the other findings, this is actually a very informative finding when generalizing to the lower educated and lower SES groups of Uganda.

In the paragraph of lines 170-174, the authors note that two data collection sites allowed participants to choose between self-administering the survey and having a staff member administer it. At three additional sites it was only allowed to be administered by staff. Although the authors analyzed and reported results for this difference, it might be advantageous address why both sites did not allow for this option.

**Grammar and Spelling:**

Line 133 should state “study” instead of “atudy”

Line 321 states that “This scenario is particularly timely in light of the **anticipated** introduction of new vaccines against SARS-CoV-2.” The authors might consider amending this to “timely in light of the introduction of…” since multiple vaccines are already being administered worldwide.

**Small items and personal feedback for the authors** (These are items that do not necessarily need to be addressed, but I wanted to convey to the authors as a personal note):

Some paragraphs are rather short, however, almost half of the paragraph is one sentence. It may do well to help the reader by cutting down some of these longer sentences to make the information more digestable. The overcomplexity of these sentences could have a negative impact on the ability of the reader to absorb and retain all the relevant points.

Although not specific to epidemic vaccination and DCE’s there is a related literature that would be of note for the authors to review or at least be aware of. Listed below are a few papers that directly investigate multiple influences on medication willingness and adherence by manipulating multiple variables related to efficacy, side effects and likelihood of taking a medication. These papers, among others in related literature, would help to support and explain some of the information that is left unexplained in the introduction. Again, this is not directly related to DCE’s or vaccines, but is related to manipulation of multiple variables in a pro-health context.

Bruce, J. M., Bruce, A. S., Catley, D., Lynch, S., Goggin, K., Reed, D., Jarmolowicz, D. P. (2016). Being kind to your future self: Probability discounting of health decision-making. *Annals of Behavioral Medicine, 50,* 297–309. <http://dx.doi.org/10.1007/s12160-015-9754-8>

Jarmolowcz, D. P., Reed, D. D., Bruce, A. S., Lynch, S., Smith, J., Bruce, J. M. (2018). Modeling effects of side effect probability, side-effect severity, and medication efficacy on patients with multiple sclerosis medication choice. *Experimental and Clinical Psychopharmacology.* 26 (6), 599-607. <http://dx.doi.org/10.1037/pha0000220>

Jarmolowicz, D. P., Reed, D. D., Schneider, T. D., Smith, J., Thelen, J., Lynch, S., Bruce, A. S., & Bruce, J. M. (2019). Behavioral economic demand for medications and its relation to clinical measures in Multiple Sclerosis. *Experimental and Clinical Psychopharmacology.*

Again, thank you to the authors for the opportunity to review your manuscript. The overall outcomes are applicable and beneficial to understanding vaccines and vaccine willingness in the current global environment.
