# Supplementary material for: What drives willingness to receive a new vaccine that prevents an emerging infectious disease? A discrete choice experiment among university students in Uganda
Source: PLoS One. 2022 May 19;17(5):e0268063. doi: 10.1371/journal.pone.0268063 (PMC9119467; doi:10.1371/journal.pone.0268063)
Supplement: Attachment — Submitted filename: Response to reviewers Feb 1 2022.docx [file pone.0268063.s005.docx]

Response to reviewers: PONE-D-21-15315

What drives willingness to receive a new vaccine that prevents an emerging infectious disease? A discrete choice experiment among university students in Uganda

Dear Reviewers,

Thank you for your thoughtful review of our manuscript entitled, “What drives willingness to receive a new vaccine that prevents an emerging infectious disease? A discrete choice experiment among university students in Uganda”. We appreciate your suggestions and have undertaken extensive efforts to fully address each comment.

In the section following, we provide a listing of each reviewer comments and a detailed description of the ways we have addressed these comments and the changes we have made to the manuscript. Reviewer text is italicized for clarity.

**Reviewer 3 Suggestions and Responses**

1. *The authors did a great job of improving this manuscript based on earlier reviewer feedback. One item remains to be addressed, which may or may not have been part of the earlier feedback from reviewers. The authors do not provide adequate theoretical context for their work. I would have appreciated some reference to the Health Belief model (see https://sphweb.bumc.bu.edu/otlt/mph-modules/sb/behavioralchangetheories/behavioralchangetheories2.html for an overview), as well as the articles on delay discounting/behavioral economics provided in the previous editorial feedback, since these concepts are directly relevant to the manuscript and this research.*

**Thank you for your positive feedback about our prior revisions. We chose to use the WHO’s behavioral and social drivers (BeSD) for vaccination framework (below), based upon the Increasing Vaccination Model, and largely derived from psychology research. We had considered the Health Belief model when developing this research. However, the Health Belief Model only captures certain elements that are outlined in the BeSD framework: the thinking and feeling domain, specifically the risk appraisal and vaccine confidence constructs. Our discussion of the BeSD and the Increasing Vaccination Model encompassess the concepts of the Health Believe Model and incorporates additional concepts that are also critically relevant to our study. To make this clear, we have noted this in the manuscript text and added a specific reference to the Health Belief model in lines 74-77:**

*“This [Increasing Vaccination] model incorporates the health belief model, including risk appraisal and vaccine confidence, into the thinking and feeling domain, while adding additional domains, including social process, and practical issues (2, 3).”*

**We agree that DCEs in general and our study in particular are grounded in behavioural economics theory, which can help explaining vaccination refusal, due to risk aversion (of side effects), over-weighting of probabilities of rare events (e.g., serious sides effects), status quo bias, or preference for the present (high discounting of future immunity).**

**However, the concept of delay discounting does not explicitly enter our study, as we are examining trade-offs made for willingness to accept, but not willingness to pay. In our study, we recognized that a vaccine for an emerging infectious disease in Uganda would almost certainly be made available free of charge as are other vaccines recommended in the country.  Discounting would find its place in modelling to identify optimized strategies.**

**To raise the importance of concepts from behavioral economics and clarify which elements we considered in designing this study with the aims presented given the cross-disciplinary nature of our study, we now note the importance of behavioural economics. The references suggested by the reviewer do not refer to concepts relevant to our study. Instead, we have identified two citations that illustrate the key points and have included those in the text lines 125-128:**

***“****DCEs measure the dominant driver of a single decision when there are multiple competing factors. Some concepts of behavioral economics, such as discounting, are explicited in DCE only if economic considerations are central to the aims of the study (26, 27), but not in a DCE on willingness to accept a vaccine.”*

2. *Finally, I would like to point out that the additional concept of Syndemics might be much more appropriate as a framing, rather than referring to epidemic as the context. A syndemic is the interaction between multiple epidemics and includes behavioral and political context. Persons make choices within those contexts, so they should not be ignored in the framing of this research.*

**Thanks for raising the question as to whether syndemic, a term which has gained much greater awareness during the COVID-19 pandemic, might be more relevant than epidemic to our study. As noted by Tsai et al (Lancet 2017), "As originally theorised, three concepts underlie the notion of a syndemic: disease concentration, disease interaction, and the large-scale social forces that give rise to them." We framed our study in terms of an epidemic because this is how the scenarios were presented to participants. We aimed to examine how participants would individually respond in the setting of a specific, acute disease outbreak-an epidemic; we did not incorporate how such an epidemic might interact with other diseases or with social and environmental factors or how these factors might shape disease dynamics within this context. Given our aims, the focus of our study on a single disease, and the emphasis we placed on examining decision-making for vaccination given the epidemiologic context alone, we believe that epidemic more accurately describes the nature of the context/scenarios presented in the study. However, we have made note of the utility of considering the broader context in future studies in the Discussion section, lines 386-389:**

*“Thus, the results may not be representative of all students in Uganda, or the willingness of other subgroups to receive a new vaccine. This may be particularly relevant when the overlay of biological and social factors in an epidemic gives rise to increased susceptibility or worse outcomes for certain groups.”*
